# Assessing the Construct Validity and Reliability of School Health Records Using the ‘Health Dialogue Questionnaire’ in the Eleventh Grade

**DOI:** 10.3934/publichealth.2016.3.470

**Published:** 2016-07-21

**Authors:** Lisbeth Kristiansen, Malin Rising Holmstrom, Niclas Olofsson

**Affiliations:** 1 Department of Nursing Sciences, Mid Sweden University, Sundsvall, Sweden; 2 Department of Health Sciences, Mid Sweden University, Sundsvall, Sweden

**Keywords:** adolescents, construct validity, factor analysis, health dialogue questionnaire, health promotion

## Abstract

**Background and aims:**

The aim for this study was to assess the construct validity and reliability of the Health Dialogue Questionnaire (HDQ^©^) for eleventh grade in school through comparison of the HDQ^©^ with Paediatric Quality Of Life Inventory (PedsQL™), Local monitoring of youth policy questionnaire (LUPP^®^), Health behaviour in Swedish school-aged children (HBSC^©^), Equal health (EH^©^) and The Swedish Survey Youth on Alcohol Consumption (SSYAC^©^).

**Methods:**

Cross-sectional samples of eleventh graders from the academic year 2009/2010 was used from the HDQ^©^ (n = 2752), the HBSC^©^ (n = 2090), the PedsQL™ (n = 666), the “LUPP^®^” questionnaires (n = 2400), EH^©^ (n = 258), and SSYAC^©^ (n = 1748) in the academic year 2009/2010. A comparison between HDQ^©^ and the different proxies was done. Exploratory and confirmatory factor analyses were performed as well as a Multitrait-Multimethod Matrix (MTMM), in order to evaluate the construct validity and reliability of HDQ^©^.

**Results:**

An average disagreement between HDQ^©^ and proxies with 10 percentages was found. Exploratory factor analysis of HDQ^©^ on the 2009/2010 sample suggested a four factor solution (girls factor solution 65% of total variance explained, and in the boys' solution 59% of total variance explained). A second sample 2010/2011 of eleventh graders were used for the confirmatory solution. Almost perfectly similar four factor solutions with were found (girls 58% of total variance explained and boys 56% of the total variance explained). Using MTMM the reliability was generally high and HDQ^©^ and showed agreeable validity.

**Discussion and conclusions:**

The HDQ^©^ questionnaire is a reliable and valid instrument for measuring eleventh graders self-reported-health in school.

## 1. Introduction

Children and adolescents spend a lot of time at schools, why these may have a major effect on their health, by teaching about health and promoting healthy behaviours. The school building and environment should be a safe and healthy place for children and adolescents. Schools work to prevent risky behaviours such as alcohol and tobacco use, inactivity or bullying, and they may also deal with specific health problems in students, such as obesity, pain management and infectious diseases.

### 1.1. What Happens for the 16 Year Old Adolescents

The physiological development is characterized with further developed cognitive ability, and the physical development focuses sexual maturity, puberty and gender differences. Girls are often two years ahead of boys in that development process. The identity development is vital, asking existential questions on meaning and life purpose. It is not unusual that the adolescents balance between being egocentric with high self-esteem, and on the other hand, lacking all confidence on the way on growing into an adult person that is more independent from family and friends in the Western view [1]. The social life is often characterized by numerous activities in sports clubs, singing, and theater etcetera with close friendship within and across gender. Friends are often first priority a substitute for family who are not, but from which an emancipation is taking place and where also ongoing experimentation of limits (smoking, alcohol, drugs) [2]–[5].

### 1.2. An Obligation to Measure Adolescents' Health

During the last years there has been an increase in the reports on adolescents, especially girl, doing mentally ill [6]. Mental health problems of perceived stress, psychological distress and deliberate self-harm, for instance, were twice as common among girls as boys [7]. Public School Health should according to the Education Act [8] monitor all students' development, preserving and improving their mental and physical health and promote healthy lifestyles among the students throughout their school years. One way to accomplish the meaning of this regulated Swedish obligation, is to perform Health dialogues (HD), which is individual meetings between the school nurse and the individual student, on the student's own perception of health (Self-reported health — SRH), where issues about lifestyle, school environment, physical, mental and social health discussed [9]–[11]. The HD haves three goals to monitor health, take measures to prevent illness and to promote health, and one part of the HD also consists of a questionnaire (HDQ©), which is relevant for this study. In order to be sure that the widely used HD, which has been used since the beginning of year 2000 in the Swedish county of Vasternorrland, is valid and reliable, it is important to investigate these reliance aspects of the HDQ^©^.

### 1.3. Research Context

The data in this study originate from the school nurses HDQ^©^ in first year in high school and the last year of ninth grade for The Swedish Survey Youth on Alcohol consumption^©^ in the county of Vasternorrland. Currently implemented, and registers each year about approximately 10,000 health consultations in the county. The county contains of seven municipals, and the region is characterized by large rural areas and few cities 243,000 inhabitants located in the middle/north of Sweden. Furthermore, the county has lower middle income, education level and proportion of residents with nonnative Swedish background compared with the national average. The population is highly homogene. The students have slightly lower average level of grades compared with the national average [12].

### 1.4. Aim

The aim was to measure the construct validity and reliability of Health dialogue questionnaire (HDQ^©^), against the following surveys; Health Behaviour in Swedish school-aged children (HBSC^©)^, the Pediatric Quality of Life Inventory (PedsQL™), Local monitoring of young policy questionnaire — LUPP®, Health on Equal terms (EH^©^), and The Swedish Survey Youth on Alcohol Consumption (SSYAC^©^).

## 2. Method

### 2.1. Sample

The validity and reliability calculation were investigated based on the data from the Health Dialogue questionnaire (HDQ©) in the first year of high school (n = 2752), 15 year old school children answering the Health behavior in Swedish school-aged children (HBSC^©^) questionnaires (n = 2090), the Pediatric Quality of Life Inventory (PedsQL™) (n = 666), the “LUPP^®^” questionnaires (n = 2400), Health on Equal terms (EH^©^) (n = 258 ), and The Swedish Survey Youth on Alcohol Consumption^©^ (SSYAC^©^) (n = 1748 ) in the academic year 2009/2010. The sample size for HDQ^©^ corresponded to 81 % girls and 82 % boys of all first grades school girls and boys in the county [12].

### 2.2. Data Collected

The text regarding the used instruments was presented as follows:

- Name;
- History, background and responsibility for the instrument;
- Which dimensions and numbers of dimensions included;
- Numbers of items and how these are sorted in to dimensions;
- How the questionnaire was distributed and handled.

#### 2.2.1. The Health Dialogue Questionnaire (HDQ^©^)

The HDQ^©^ was born out of clinical practice where school nurses sought a structured and systematic work approach. The County Council and the seven municipalities reached consensus on a systematically structured approach to the collection of student's health data [5].

The HDQ^©^ consisted of a structured questionnaire with 38 questions covering three dimensions of health a) physically, b) mentally, and c) socially. a) The physical dimension included six items; nutrition, physical activity, school environment, allergies, asthma, and sleep habits. b) The mental dimension included three items; mental mood (anxiety, feeling sad or depressed), bulling, having ability to concentrate and work in school. While, c) the third dimension was related to social including two items, leisure and drug use.

HDQ^©^ used a population based sampling procedure and it represented a cross-sectional image of the student's SRH regarding physical, mental and social health. The HDQ^©^ was annually offered to all students at four occasions: in preschool, in fourth grade, in seventh grade, the first year of high school, and follows the student's development and growth from 6 to 16 years old, and therefore repeat the content with age appropriate questions [10],[11],[13].

The school nurses who conduct the HDQ^©^ was situated at the school and were responsible for the data collection at each separate school i.e. distribution of written as well as verbal information to all students and parents regarding voluntary participation and the registration of each HDQ^©^. The school nurses were also responsible for the individual feedback to the students. Provided that the parents had given their written consent, the school nurses afterwards registered the questionnaires.

#### 2.2.2. Health Behavior in Swedish School-aged Children (HBSC^©^)

The HBSC^©^ was a part of the WHO global survey, and since 1985/1986, this study has taken place around the world every four years, most recently in over 40 different countries. The international standard questionnaire enables the collection of common data across participating countries and thus enabled the quantification of patterns of key health behaviours, health indicators and contextual variables. School children aged 11, 13 and 15 were asked to answer different health questions during their ordinary school classes. The Swedish National Institute of Public Health has been assigned by the Government to monitor the development of determinants of health, and the HBSC^©^ represented a cross-sectional image of student's health in that assignment. The data also allowed cross-national comparisons to be made and, with successive surveys, trend data analysis was also possible [14].

The HBSC^©^ consisted of a structured questionnaire with 82 questions covering four dimensions of health behaviour among school aged children. The dimensions were ‘Self-rated health and general well-being’, ‘Lifestyle’, ‘Social relationships’ and ‘School’.

The sampling method for participating school children was carried out in a two-step cluster design. First, a national representative cluster of schools was randomly selected. Second, a selection of schools or classes in each grade was included in the study, with at least 1500 school children in each grade; data consisted of school children 15 years old from the academic year 2009/2010. The cluster of schools differs for each occasion the HBSC^©^ is conducted.

Furthermore the HBSC^©^ was a school-based survey with data collected through self-completion questionnaires administered in the classroom by teachers. The HBSC^©^ did not include individual contact, dialogue or feedback concerning the questionnaires with the students. The teacher was responsible for distribution of information concerning the study, such as parental agreement and student´s voluntary participation.

The HBSC^©^ survey was mostly analysed and reported at a national level and used in the research context.

#### 2.2.3. Pediatric Quality of Life Inventory (PedsQL™)

The PedsQL™ measurement model was a modular approach to measuring health-related quality of life in children and adolescents. The PedsQL™ consisted of brief, practical, generic core scales suitable for use with healthy school and community populations, as well as with paediatric populations with acute and chronic health conditions. PedsQL™ condition-specific modules complement the generic core scales for use in designated clinical populations. Accordingly, the PedsQL™ consisted of developmentally appropriate forms for children ages 2–4, 5–7, 8–12, and 13–18 years. Paediatric self-report was measured in children and adolescents ages 5–18 years. The PedsQL™ was not used on a regular base in school environment, but mostly used in the research context [15].

The multidimensional PedsQL™ Generic Core Scales encompassed the essential core domains for paediatric health related quality of life measurement: a) ‘Physical Functioning’ (8 items), b) ‘Emotional Functioning’ (5 items), c) ‘Social Functioning’ (5 items), and d) ‘School Functioning’ (5 items).

The questionnaires were distributed by the school nurses in one of the municipalities in the county at the same time as HDQ^©^ was distributed and to the same individuals. In this study we regarded the PedsQL™ as a Golden Standard [16].

#### 2.2.4. Local Monitoring of Young Policy Questionnaire (LUPP^®^)

The LUPP^®^ (a local follow-up of youth policy) was a cross-sectional survey that enabled municipalities to gather knowledge on the living situation of young people, as well as information on their experiences and opinions [17]. In order to provide the basis for policy decisions, the Swedish Youth Board and the country's municipalities have performed a survey to produce knowledge of youth in Sweden yearly since 2001. The Swedish Agency for Youth and Civil Society has developed the LUPP^®^ survey in consultation with municipal representatives and researchers. The survey was adapted for different age groups: young people at compulsory school aged 13–15; young people at upper secondary school aged 16–18; and young adults 19–25 years old.

The LUPP^®^ survey contained approximately 80 questions that were divided into different modules with questions concerning the perception of influence and democracy, wellbeing school, leisure activities, work, health and future plans.

The LUPP^®^ survey could be carried out electronically or on paper, by the latter age group, a web-based survey, while the younger age group typically use questionnaires implemented during a lesson hours in school by teachers. Distribution is in line with the HBSC^©^ meaning no individual contact, dialogue or feedback concerning the questionnaires with the students. LUPP^®^ survey was analysed and reported at a municipal and national level and used in the research context.

#### 2.2.5. Health on Equal Terms (EH^©^)

Due to peoples' health inequalities and in order to be able to survey the development in the Swedish populations' health, health habits and living conditions health in a wide sense. The EH^©^ was constructed by the Swedish national board of public health in cooperation by the national statistical bureau, Statistics Sweden, as a survey distributed nationally to approximately 20,000 inhabitants annually since 2004 [18]. EH^©^ covered questions related to physical and mental health, consumption of pharmaceuticals, contact with healthcare services, dental health, living habits, financial conditions, work and occupation, work environment, safety and social relationships. Further, the county councils have been provided with an opportunity to add regionally relevant questions.

EH^©^ consisted of a structured questionnaire with 81 questions that had several answer options, which generated a total of 143 questions, covering seven dimensions of health and living conditions among 16 to 84 year old people. The dimensions were ‘Self-rated health’, ‘Living conditions’, ‘Economical circumstances’, ‘Work and preoccupation’, ‘Security and Social relationships’.

The sampling method for participating students was carried out using a representative design, where a random sample was drawn. This was again randomly stratified on a county council level from a total population of 194,000 people. The participants in different year classes, including the 16 year old, were randomly selected. The questionnaire was administered by the Statistics Sweden. The participants were given the opportunity to answer on paper or by the web. Two reminders were given. The data consisted of students 16 years old from the academic year 2009/2010.

#### 2.2.6. The Swedish Survey Youth on Alcohol Consumption (SSYAC^©^)

The Swedish Council for Information on Alcohol and Other Drugs (CAN) is a non-governmental organization with regional and local representation in the whole country. It has followed the drug trends in Sweden and informed the public and educated professionals on alcohol and other drugs. Since 1971 CAN has annually measured drug consumption of adolescents in the ninth and eleventh grade in order to follow the development of drug habits by using the SSYAC^©^ [19]. The survey is distributed to a nationally representative sample of approximately 300 school classes. Though, the county councils have opportunities to distribute the SSYAC^©^ to all pupils in the ninth and eleventh grade, which was done in Vasternorrland.

The SSYAC^©^ covered five dimensions of drug habits, consumption and attitudes a) Tobacco, b) Alcohol, c) Tranquilizers, d) Illegal drugs, and e) Attitudes. It consisted of a structured questionnaire with 76 questions that had several answer options, covering questions on drug use frequency, amount, type, and how they gained access to the different drugs among the fifteen and seventeen years old.

The sampling method was carried out as a total population survey. The county council in Vasternorrland chose to distribute the SSYAC^©^ to total population of all 132 schools. The survey was distributed by the teachers at the school during ordinary class hours and the students were requested to answer during school time.

### 2.3. Statistical Analyses

#### 2.3.1. Statistical Analysis of the Validation

Assessing the usefulness of school health records demands a structured approach to validity and reliability testing. In our analysis scheme three major steps were taken. By conducting descriptive comparisons on percentage of positive answers on the five different surveys population proxies with the HDQ^©^, we firstly, measured the convergent validity as it is important aspect of construct validity. All answers are from the comparable proxy samples, which expect us to reveal a high degree of equivalence between the HDQ^©^ and the surveyś items percentage score, which is a sign of good convergent validity. The researchers used comparable proxy samples as the aim was to find a robust theoretical construct model, irrespectively context [20].

#### 2.3.2. Statistical Analysis for HDQ^©^

In the second major step, was an exploratory factor analysis, conducted to assess the results from the HDQ^©^. In the analysis we chose to use the items that were shown with at least four available proxies. Before conducting the analyses we excluded one item (Being bullied) as this is a dichotomised item, which do not add meaning in the factor analysis. Varimax rotation with Kaiser's normalisation were performed separately for both sexes. This resulted in twelve factors, but eight factors were excluded as they did not contribute to a useful interpretation of the structure meaning [21],[22]. Four firm factors remained for both sexes, though not similar, and they accounted for approximately 57 per cent of the cumulative variances (for girls 57.9 % and for boys 56.1 %). In the result section the names (groups of items) of the factors and their eigenvalue and variance explanation is shown.

To perform a confirmatory analysis we have included one other HDQ^©^ sub-sample from the academic year 2009–2010. The analysis was also when conducted based on the four factor model. Here, we compared the study sample's answers to HDQ^©^ the academic year 2009–2010 on total county level with another total country sample from the academic year 2010–2011 in order to test the stability.

#### 2.3.3. Multitrait-Multimethod Matrix

In our third major step we applied the Multitrait-Multimethod Matrix (MTMM) in this study as an approach to assess both the reliability (internal consistency) using Cronbach's Alpha [23] and the construct validity of a set of measures also calculating the convergent validation parts [24],[25].

#### 2.3.4. Ethics

This study followed the ethical principles recommended by the Research Council and was approved by the Ethics Committee at the Medical Faculty, Umea University, to be evaluated the (Dnr 2008-122M).

## 3. Results

The HDQ© was salutogenic, and as being so, the percentages of positive answers are presented in table 1 (girls) and table 2 (boys). Measuring the concurrent validity could be done by comparing the percentages emanating from the HDQ^©^ with similar questions from different but resembling questionnaires HBSC^©^, LUPP^®^, EH^©^, SSYAC^©^, and PedsQL™. The items in table 1 and 2 that differed least between HDQ^©^ and the proxies were items that concerned alcohol, smoking, snuff and self-reported health. There was an overall high level of fit between HDQ^©^ and the proxies. Describing and analysing the percentages in Table 1 and table 2, the percentages were averagely missing similarity with 10 percentages within the girls (Table 1) and 10 percentages in the boys (Table 2); the items “Sleep” in the girls sample and “Nervousness” in the boys sample, differ mostly between HDQ^©^ and the population proxies.

**Table 1.** Percentages of positive answers from the 11^th^ grade HDQ^©^ and their population proxy scores (girls; n = 1329).

Girls	HDQ©	HBSC©	SSYAC©	EH©	LUPP®	PedsQL™
Never tried alcohol	33		31		30	
Never tried drugs	96		96		92	
Never tried smoking	57	48	42	93	62	
Never tried snuff	76	75	56	97	81	
Feeling low/sad	54	46		41		62
Irritable/bad tempered	40	30				64
Nervousness	76	51		63		79
Back/neck/shoulder ache	52	68		60		58
Headache	51	48		57	53	
Stomache ache	64	63		87	65	
Always concentrate	74			82	91	65
Feeling pressured by schoolwork sometimes or less	70	47		84	25	71
Not bullied	97	91	82	67		99
Rather good or better school satisfaction	93	87	76			
Rather good or better self-reported health	84	97		86	65	
Sleep	76	30		78	46	78
Someone to talk with	94		82	90		
Daily physical activity	24	5		38	14	
Drinking soft drinks once a week or less	19	12				
Eating breakfast every schoolday	63	58			56	
Less than 1 hour computer a day	30	30				
Less than 1 hour TV a day	30	40				

Note: Only HDQ^©^ items with comparable proxies are shown (Concurrent validity).

**Table 2.** Percentages of positive answers from the 11^th^ grade HDQ^©^ and their population proxy scores (boys; n = 1423).

Boys	HDQ^©^	HBSC^®^	SSYAC^®^	EH^®^	LUPP^®^	PedsQL™
Never tried alcohol	33		40		34	
Never tried drugs	93		98		87	
Never tried smoking	57	55	42	96	64	
Never tried snuff	60	63	48	91	66	
Feeling low/sad	86	77		69		88
Irritable/bad tempered	60	49				79
Nervousness	94	66		82		90
Back/neck/shoulder ache	67	76		80		77
Headache	78	73		78	76	
Stomache ache	76	82		91	74	
Always concentrate	72			93	93	70
Feeling pressured by schoolwork sometimes or less	84	68		96	57	77
Not bullied	99	90	82	81		99
Rather good or better school satisfaction	93	93	79			
Rather good or better self-reported health	91	98		91	76	
Sleep	78	43		82	57	80
Someone to talk with	95		84	85		
Daily physical activity	28	10		45	19	
Drinking soft drinks once a week or less	15	10				
Eating breakfast every schoolday	62	70			53	
Less than 1 hour computer a day	30	38				
Less than 1 hour TV a day	30	37				

Note: Only HDQ^©^ items with comparable proxies are shown (Concurrent validity).

### 3.1. Exploratory Factor Analysis

Exploratory factor analysis of HDQ^©^ on the 2009/2010 sample suggested a four factor solution. In the girls factor solution 65% of total variance explained, and in the boys' solution 59% of total variance explained. Firstly the girls' solution is described, and secondly and lastly the boys' solution. The first factor termed “Mental health” consisted of positive loadings of “feeling low or sad”, “nervousness”, and negative loading of “rather good or better self-reported health”. The second factor (Physical health) consisted of positive loadings of “back, neck, and/or shoulder ache”, “headache”, and “stomach ache”. The third “School” factor consisted of positive loading for “feeling pressured by school sometimes or less”, and negative loadings in “sleep” and “always concentrate”. The fourth and last factor (Behaviour) included positive loadings of “never tried smoking”, “never tried snuff” and negative loading for “daily physical activity” (Table 3). Two differences were found in the boy's four factor solution. In the “Physical health” factor “feeling pressured by schoolwork sometimes or less” loaded positively in the “Physical factor” rather than in the “school” factor as for the girls. Instead of the schoolwork pressure item in the school factor the boys' “daily physical activity” load positively in that particular factor (Table 4).

**Table 3.** Four factor solution for comparing two HDQ^©^ samples from different academic years (Girls).

2009/2010 girls	Factor 1	Factor 2	Factor 3	Factor 4
**Mental health**				
*Feeling low/sad*	0.793	0.212	0,201	0.041
*Nervousness*	0.659	0.202	0.263	0.018
*Rather good or better self reported health*	−0.533	−0.235	−0.392	−0.081
**Physical health**				
*Back/neck/shoulder ache*	0.085	0.587	0.257	0.116
*Headache*	0.142	0.479	0.217	0.086
*Stomache ache*	0.287	0.463	0.143	0.145
**School**				
*Feeling pressured by schoolwork sometimes or less*	0.148	0.138	0.445	0.001
*Sleep*	−0.235	−0.214	−0.442	−0.215
*Always concentrate*	−0.169	−0.197	−0.441	−0.103
**Behavior**				
*Never tried smoking*	0.070	0.116	0.123	0.612
*Never tried snuff*	−0.048	0.195	0.013	0.546
*Daily physical activity*	−0.100	0.055	−0.027	−0.114
2010/2011				
**Mental health**				
*Feeling low/sad*	0.809	0.208	0.252	0.063
*Nervousness*	0.635	0.211	0.242	0.054
*Rather good or better self reported health*	−0.518	−0.297	−0.435	−0.097
**Physical health**				
*Headache*	0.180	0.623	0.184	0.098
*Stomache ache*	0.323	0.511	0.089	0.146
*Back/neck/shoulder ache*	0.102	0.488	0.240	0.141
**School**				
*Always concentrate*	−0.147	−0.079	−0.526	−0.144
*Sleep*	−0.176	−0.273	−0.460	−0.147
*Feeling pressured by schoolwork sometimes or less*	0.212	0.172	0.433	−0.023
**Behavior**				
*Never tried smoking*	0.065	0.040	0.070	0.761
*Never tried snuff*	−0.013	0.133	0.063	0.558
*Daily physical activity*	−0.082	−0.104	−0.065	−0.146

**Table 4.** Four factor solution for comparing two HDQ^©^ samples from different academic years (Boys).

2009/2010 boys	Factor 1	Factor 2	Factor 3	Factor 4
**Mental health**				
*Feeling low/sad*	0.804	0.139	0,105	0.056
*Nervousness*	0.671	0.189	0.055	0.024
*Rather good or better self reported health*	−0.490	−0.260	−0.356	−0.024
**Physical health**				
*Headache*	0.092	0.572	0.161	0.020
*Stomache ache*	0.182	0.534	0.067	−0.003
*Back/neck/shoulder ache*	0.096	0.468	0.138	0.164
*Feeling pressured by schoolwork sometimes or less*	0.215	0.271	0.171	0.050
**School**				
*Sleep*	−0.201	−0.165	−0.696	−0.0765
*Always concentrate*	−0.132	−0.116	−0.332	−0.172
*Daily physical activity*	−0.005	0.042	0.151	0.052
**Behavior**				
*Never tried smoking*	0.131	0.060	0.186	0.646
*Never tried snuff*	−0.047	0.062	0.113	0.604
2010/2011				
**Mental health**				
*Feeling low/sad*	0.789	0.150	0.023	0.193
*Nervousness*	0.664	0.201	0.027	0.079
*Rather good or better self reported health*	−0.487	−0.245	0.061	0.448
**Physical health**				
*Headache*	0.083	0.578	0.081	0.156
*Stomache ache*	0.184	0.528	0.036	0.062
*Back/neck/shoulder ache*	0.140	0.423	0.152	0.163
*Feeling pressured by schoolwork sometimes or less*	0.224	0.253	0.051	0.211
**Behavior**				
*Never tried smoking*	0.123	0.044	0.760	0.090
*Never tried snuff*	−0.042	0.054	0.548	0.083
*Daily physical activity*	0.006	0.085	0.170	0.098
**School**				
*Sleep*	0.165	0.173	0.173	0.624
*Always concentrate*	0.119	0.138	0.210	0.338

### 3.2. Confirmatory Factor Analysis and MTMM

Intending to confirm the four factor solution, a confirmatory factor analysis were performed. A second sample 2010/2011) of eleventh graders were used. For the girls a perfectly similar four factor solution with 58% of total variance explained were found. In the boys' confirmatory solution “daily physical activity” changed factor from “School” to “Behaviour” and the two factors (“School” and “Behaviour”) change place as explaining factors. In the boys' solution in the 2010/2011 sample, 56% of the total variances were explained.

As could be seen in the reliability diagonal the internal consistency measured by Cronbach's alpha, was generally high in both HDQ^©^ and PedsQL™ (physical health 0.65, mental health 0.76, behaviour 0.48, and school 0.53). Correlations between measures of the same trait measured using different methods should be understood as validity, i.e. the correlations between Physical health in HDQ^©^ correlated 0.46** with Health in PedsQL™, while HDQ^©^ mental health correlated 0.78** with PedsQL™'s Emotional dimension, and 0.37** Social dimension in PedsQL™. Behaviour in HDQ^©^ correlated 0.12* with Behaviour in PedsQL™, and 0.61* with School. At the same time School in HDQ^©^ correlate 0.35** to school in PedsQL™ (Table 5).

The essentially evidence of convergent validity is when the validity coefficient are higher than values lying in its column and row in the same method block (i.e. HDQ^©^ block and PedsQL™ block). The total scale correlate 0.76** between HDQ^©^ and PedsQL™, and the scale relation and the dimensions. Many (almost half of the values) of the correlations in table 5 meet this criterion when comparing between dimensions of the different blocks (Table 5).

**Table 5:** Using a Multitrait-Multimethod Matrix reliability diagonal showed by Cronbach's alpha and convergent validity by total scale correlations.

		PedsQL™						HDQ^©^			
		Health		Emotion		Social	School	Physical health	Mental health	Behavior	School
PedsQL™	Health	0.75									
	Emotion	0.54**		0.78							
	Social	0.42**		0.42**		0.72					
	School	0.59**		0.60**		0.37**	0.75				
HDQ^©^	Physical health	0.46**		0.52**		0.20**	0.51**	0.65			
	Mental health	0.46**		0.78**		0.37**	0.54**	0.51**	0.76		
	Behavior	0.35**		0.24**		0.12**	0.61*	0.20**	0.25**	0.48	
	School	0.45**		0.60**		0.24**	0.35*	0.50**	0.42**	0.25**	0.53

* indicates significant associations at 95 % level and ** indicates sign ass. at 99%.

## 4. Discussion

### 4.1. Result Discussion

The objective for this study was to estimate validity and reliability for HDQ^©^ for eleventh grades. The results suggest good convergent validity and good reliability. In the first step, we found a good concurrent agreement validity, a part of the criterion validity, as only a ten percentage difference between the HDQ^©^ and the proxies were seen. We did not consider any of the chosen reference questionnaires, the population proxies, as a Golden Standard [16] in this step. For construct validity testing, we hypothesised two gender based models for exploratory and confirmatory, where we posed a four factor model solution for both. This was tested using two different samples from different academic years. We constructed the same solution for the two girl sample and a highly equivalent solution for the boys. For the girls these models explained a good and satisfying level of the variances (exploration = 65%, confirmation = 59%), which also was the case regarding the boys' models (exploration 58%, confirmation = 56% variances) explained. This means a high construct validity.

When using Multitrait-Multimethod Matrix on HDQ^©^, the PedsQL™ served as a Golden Standard. Expect from the correlation between “Behaviour” in HDQ^©^, which correlated 0.12* with Behaviour in PedsQL™, pointing at possible item overlap related to how the respective questionnaire decide to categorized them, where is good level of reliability. PedsQL™. Further, the essentially evidence of convergent validity is when the validity coefficient are higher than values lying in its column and row in the same method block (i.e. HDQ^©^ block and PedsQL™ block) [24]. The total scale correlate 0.76** between HDQ^©^ and PedsQL™, and the scale relation and the dimensions. Many (almost half of the values) of the correlations in table 5 meet this criterion when comparing between dimensions of the different blocks. Generally, there were good convergent validity.

### 4.2. Methodological Consideration

First of all, we would like to further evolve the argumentation of the chosen validity and reliability testing approach for the HDQ^©^ Self-reported health of youngsters. In some traditions a test-retest procedure is preferable and regarded a standard solution. However, only conceiving reproducibility as the extent to which similar results are obtained when an instrument is administered on repeated occasions to stable subject is not enough [26]–[28]. When it comes to develop and testing an instrument measuring health, especially self-reported health, we argue that a test-retest approach has limited scientific value [29]. The experience of health, changes quite rapidly, not at least regarding children and young people. A bad lecture might colour their global view on health the rest of the day. We therefore chose to compare with other questionnaires. This choice did not prevent us from having other methodological challenges. An overall approach in HDQ^©^ is its foundation on “salutogenic or positive health concept”, which impacts the way the questions are posed to the children and the youngsters, for instance, a question regarding sleep is phrased: *“I sleep well”.* This is not the case of all the questionnaires that served as comparison or population proxies in this study, leading to different ways of posing sleep questions. The wording on sleep in the HBSC^©^ is: *“How often do you have problems sleeping”.* This, of course, is a consideration to bear in mind.

Another important consideration is that the obvious differences concerning the questionnaire administration procedures, where the HDQ^©^ involves individual contact and dialogue with the school nurse, did not have any higher impact on the reliability and the validity (the differences were 10 percentage though out the results) [25],[29]. It could be stressed again that the aim was to measure the construct validity and reliability of Health dialogue questionnaire (HDQ^©^), against the following surveys; Health Behaviour in Swedish school-aged children (HBSC^©)^, the Pediatric Quality of Life Inventory (PedsQL™), Local monitoring of young policy questionnaire — LUPP®, Health on Equal terms (EH^©^), and The Swedish Survey Youth on Alcohol Consumption (SSYAC^©^). One important aspect of construct validity is to connect the tested questions to similar questions measuring the same construct with similar or different contexts, and comparable samples and find them to be related [20].

An important aspect of all utilised questionnaires is the level of “reliable answers”, we observed that the more concrete the phrasing of questions were, including if the questions were posed in absolute terms in yes or no answers, the more reliable they seemed. As a consequence, we chose to exclude the HDQ^©^ items that asked for specific about knowledge about some schoolmate being or oneself being bullied. There are even items about whom was the perpetrator. Some questions from HDQ^©^ were related to eating habit, and also on whether or not the students performed paid worked outside their school hours as it was not meaningful to show them as they lacked equivalents in the proxies. On the other hand, the HDQ^©^ did not have any questions related to how well the young people were getting along with each other, which the PedsQL™ do.

### 4.3. Clinical Implications and Conclusion

From a methodological perspective, the natural chose when working with measuring student's SRH, is to include HDQ^©^ due to its' good validity and reliability at least in a Swedish population. The first step is taken towards a language validation of the HDQ which is needed to test validation and reliability internationally.
